# Biomechanical Test of a New Endoprosthesis for Cylindrical Medullary Canals in Dogs

**DOI:** 10.3389/fvets.2022.887676

**Published:** 2022-06-30

**Authors:** Rosa Mendaza-DeCal, Yolanda Ballesteros, Salvador Peso-Fernandez, Eva Paz, Juan Carlos del Real-Romero, Jesus Rodriguez-Quiros

**Affiliations:** ^1^Animal Medicine and Surgery Department, Veterinary Faculty, Complutense University of Madrid, Madrid, Spain; ^2^ABAX Innovation Technologies, Villanueva de la Cañada, Madrid, Spain; ^3^Institute for Research in Technology/Mechanical Engineering Department, Universidad Pontificia Comillas, Madrid, Spain

**Keywords:** biomechanical test, patient-specific implant, FDM, PEEK, exo-endoprosthesis, *ex vivo*, tibia, canine

## Abstract

Exo-endoprosthesis is a limb salvage procedure for animals, although only expensive metal devices have been described. Now-a-days, new materials for this type of implant could be considered due to novel and affordable manufacturing techniques. However, a factor of safety (FoS) should be considered. There are kinetic and kinematic studies of canine natural gaits, which can be used to establish an FoS for mechanical tests for new non-metallic devices. Polyetheretherketone (PEEK) is used in different specialties in human medicine. Its mechanical properties (and its close mechanical stiffness to that of bone) make this polymer an alternative to metals in veterinary traumatology. PEEK could also be used in 3D printing. The suitability of a novel inner part of an exo-endoprosthesis manufactured by fuse deposition modeling (FDM) was presented in this study for long canine bones. Mechanical characterization of 3D-printed PEEK material and *ex vivo* mechanical tests of a customized endoprosthesis were performed to address it. Young's modulus of 3D-printed PEEK suffered a reduction of 30% in relation to bulk PEEK. Customized 3D-printed PEEK endoprostheses had promising outcomes for the tibiae of 20 kg dogs. Pure compression tests of the non-inserted endoprostheses showed a maximum force of 936 ± 199 N. In the bending tests of non-inserted endoprostheses, the PEEK part remained intact. Quasistatic mechanical tests of bone-inserted endoprostheses (compression-bending and pure compression tests) reached a maximum force of 785 ± 101 N and 1,642 ± 447 N, respectively. In fatigue tests, the samples reached 500,000 cycles without failure or detriment to their quasistatic results. These outcomes surpass the natural weight-bearing of dogs, even during a galloping pace. In conclusion, the 3D-printed PEEK part of the endoprosthesis for an exo-endoprosthesis can withstand loading, even during a galloping pace.

## Introduction

Exo-endoprosthesis is a limb salvage procedure in humans and, to a lesser extent, in animals (1–5). Because the implant is inserted into the medullary canal of a long bone, its advantages are no pain, no delay in load transfer, energetic efficiency, and good proprioception. However, complications have been reported, such as infection, skin breakdown, aseptic loosening, device failure, and avulsion (2, 5, 6). The two last complications can be related to stress-shielding phenomena (7). Therefore, aseptic loosening and stress-shielding are related to the higher stiffness of metals as compared to bone (7, 8) and metallic debris. A rigid material is reluctant to deform and difficult to adapt when it is inserted in a flexible object, like the medullary canal of a bone. So perfect fitting of the rigid material to the bone cannot be expected. This mismatch produces a lack of stress transmission between rigid material and bone, which is one of the reasons for stress-shielding. In the same way, it is the flexible material, the bone, which is deformed because of the rigid material in case of irregular stress transmission. Thus, these phenomena are related to the chosen material of a medical device, and their effect could be reduced by selecting other materials with a stiffness closer to the bone. Now-a-days, all veterinary implants are individualized, and made in metal. These characteristics make them an expensive solution that few owners can afford for their pets.

Seeking price reduction and avoiding the aforementioned complications related to metal stiffness, other materials could be considered for implants. The stiffness is directly related to the elastic properties of a material, such as Young's modulus, tensile strength, and bending strength among others (9). Materials with a much higher Young's modulus, such as metals, when compared with bone, could be prone to stress-shielding of the host bone due to their rigidity (7, 10). Therefore, a non-metallic material with bone-like elastic properties could be considered to reduce the aseptic loosening and stress-shielding of a medical device on a bone.

Polyetheretherketone (PEEK) is one of those alternative non-metallic materials for medical devices. PEEK is a thermoplastic of the polyaryletherketone family that is generally used for different specialties in human medicine, such as neurosurgery, traumatology, and dental and craniomaxillofacial surgery (11, 12). PEEK is also bioinert in the physiological environment and has good biocompatibility (11). It is one of the polymers with good mechanical properties, such as its weight-bearing and strength properties (11). Furthermore, its stiffness is closer to that of bone than to that of metal (11). All of these specific characteristics make it a candidate for the avoidance of stress-shielding as is assessed on ovine or canine specimens in experimental studies for different traumatological devices (11–14).

Now-a-days, thanks to additive manufacturing (AM), quite a range of materials can be considered for manufacturing small productions of final devices with a minimum cost (15, 16). There are different technologies in the field of AM, although only direct metal laser sintering (DMLS) has been reported in papers in the field of veterinary medicine as a manufacturing method for endoprosthesis or intraosseous implants (5, 6, 17, 18). This type of technology is cheaper than traditional manufacturing (15). However, it is still quite expensive when compared with fused deposition modeling (FDM) (16). Currently, there are a few patents for 3D-printed exo-endoprostheses that are manufactured in FDM, such as ES2736410A1 (19) or US10925755B2 (20). Nevertheless, the mechanical properties of polymers manufactured by FDM should be evaluated, as these properties are usually inferior to those obtained by the injection molding process (21–23). Thus, preliminary tests are recommended with tensile test samples of the studied polymers. Tensile test samples are shaped according to ISO 527-1 (24).

To assess a new material for a load-bearing device, a factor of safety (FoS) should be determined to endorse the feasibility of the material for that activity and situation. Currently, the FoS and working load have not been determined for veterinary limb salvage devices. Working load is the applied load on the device during the activity. FoS is a ratio defined for any mechanical device to avoid failure during its work, expressed as the ratio of the maximum load (failure load) to the working load. As a working load, these devices will withstand different body weights (BWs) according to the type of pace and moment of the stance phase (25–27) during the gait cycle. Each canine pelvic limb bears approximately 0.44 times its BW during walking (28, 29), around 0.71 times its BW during trotting (25), and 1.5 times its BW while dogs are in a steady gallop (26, 30). To compare the aforementioned weight-bearing forces, data have been normalized to the BW of animals using the BW equation described by Krotscheck et al. (31). Hence, these values could be considered as working load for determining the FoS for *ex vivo* mechanical testing of new load-bearing implants. Currently, published quasistatic and dynamic mechanical tests of other veterinary implants (5, 32, 33) just expected device failure without relating it to an actual canine weight-bearing force. In addition, the mechanical tests will need the placement of the bone specimens as close as possible to the real positions during canine paces. Kinematic studies can be used as a reference for this issue. A range of 126° to 147.2° is collected for a stifle joint during walking at a stance stage (34), while a range of 107° to 159° is registered at trotting, galloping, and acceleration (27, 30). These data give an average stifle angle of 135°, which is the assumed value of this joint for dogs in veterinary medicine. In a dynamic test, the stride per second of each pace can be used as a reference for adjusting frequency values (35). Strides per second range approximately from 3 to 1 depending on galloping to walking pace, respectively.

The aim of this study is to determine if PEEK material in contact with the bone could be a possible weight-bearing option for canine patients during their regular gaits. This work describes initial *ex vivo* research on the use of a PEEK sleeve to interface between the bone and inner part of an exo-endoprosthesis in the context of limb-sparing surgery. The 3D-printed endoprosthetic element was tested by new loading protocols to mimic normal canine loads during walking, trotting, and galloping. To our knowledge, no evidence of mechanical tests was found for other exo-endoprostheses in veterinary medicine.

## Methods

### Endoprosthesis Design

**Figure 1:**
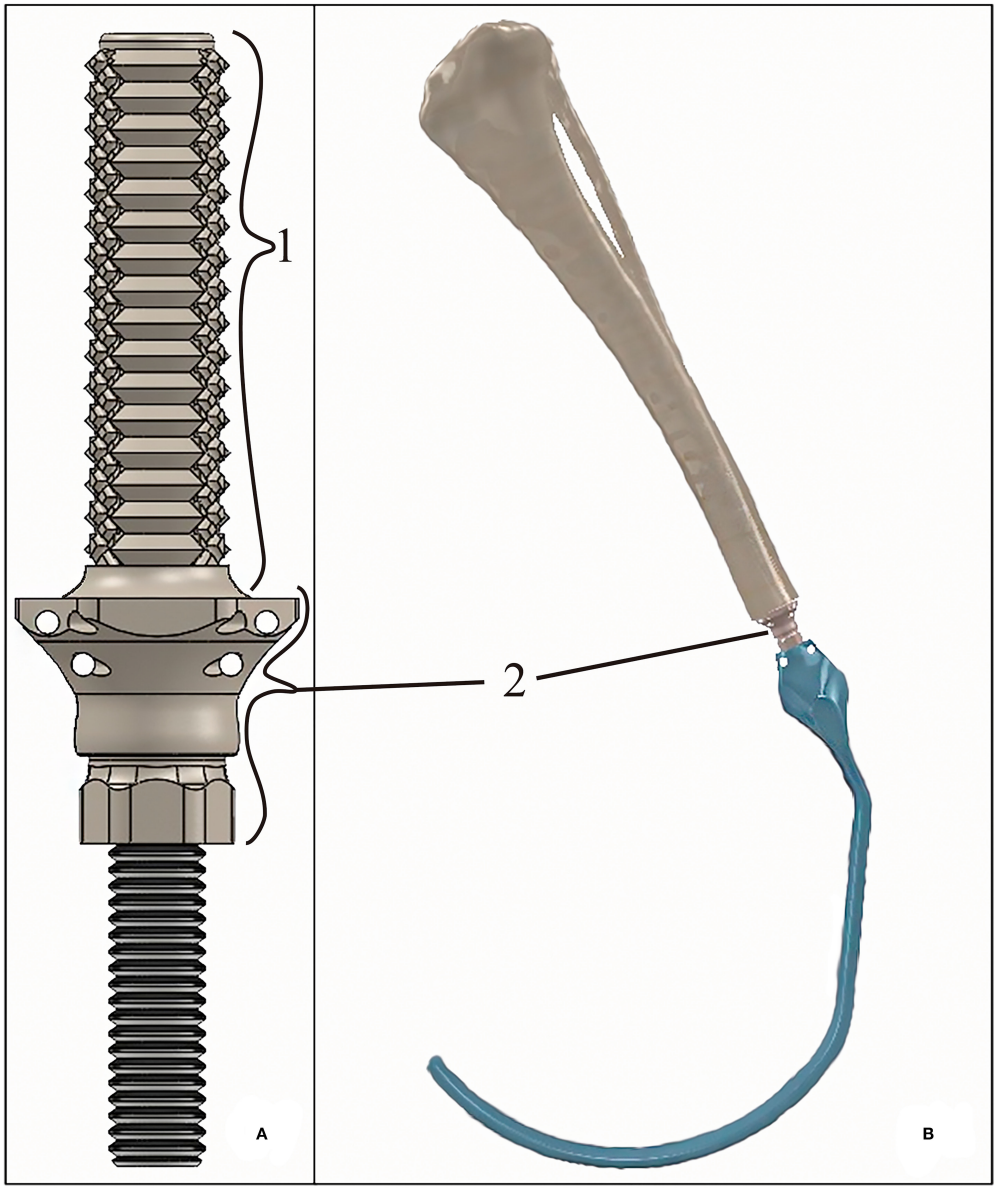
**(A)** 3D-rendered model of the tibial endoprosthesis and **(B)** 3D-rendered assembly of the exo-endoprosthesis inserted into a tibia. The exo-prosthesis part (blue) is an example of the ideal construct. Where (1) represents the stem; and (2) represents an approximation base- “umbrella” section.

The assessed endoprosthesis was patented by Mendaza et al. (19) and was composed of two main elements: (i) the PEEK sleeve, composed of a base, “umbrella”, neck, and stem (36), which will be in contact with bone tissue, and (ii) surgical metal threaded rod, made of AISI 316 austenitic stainless steel, which will be attached to an exoprosthesis (Figure 1).

The PEEK sleeve of the endoprosthesis was designed with a CAD program (SolidWorks, SolidWorks Corp., Waltham, MA). The external diameter of the stem was 0.40 mm larger than the drilled medullary canal of a tibia (9 mm). Thus, all external diameters of the stems measured 9.40 mm. The diameter of the inner cavity of the PEEK sleeve was 6 mm just like the diameter of the threaded rod. This cavity was slightly reduced at the level of the stem for applying extra-radial compression to the bone, such as that described by Mendaza-DeCal et al. (36).

### 3D Printing Material

Tibia endoprostheses and tensile and bending test samples were printed in PEEK (3D4Makers, Amsterdam, Netherlands). Filaments were stored in a special ziplock multi-layered bag with a special EVOH barrier film and silica desiccant sachets provided by the filament supplier. Otherwise, following the recommended protocols in the literature (22) and by filament manufacturers, spools were dried at 150 °C for 3 h in a dry heat oven before printing with PEEK.

### 3D Printing Manufacturing Parameters

Tensile and bending test samples and endoprostheses were printed by FunMat HT (INTAMSYS, Shanghai, China) based on FDM technology, with a build volume of 260 mm × 260 mm × 260 mm (x, y, and z). The printer has a hardened steel nozzle of 0.4 mm diameter. This printer reaches a nozzle temperature of up to 450 °C, bed temperature of up to 160 °C, and chamber temperature of up to 90 °C. Before printing, 3D virtual models were sliced by Simplify 3D software (Cincinnati, USA).

**Figure 2:**
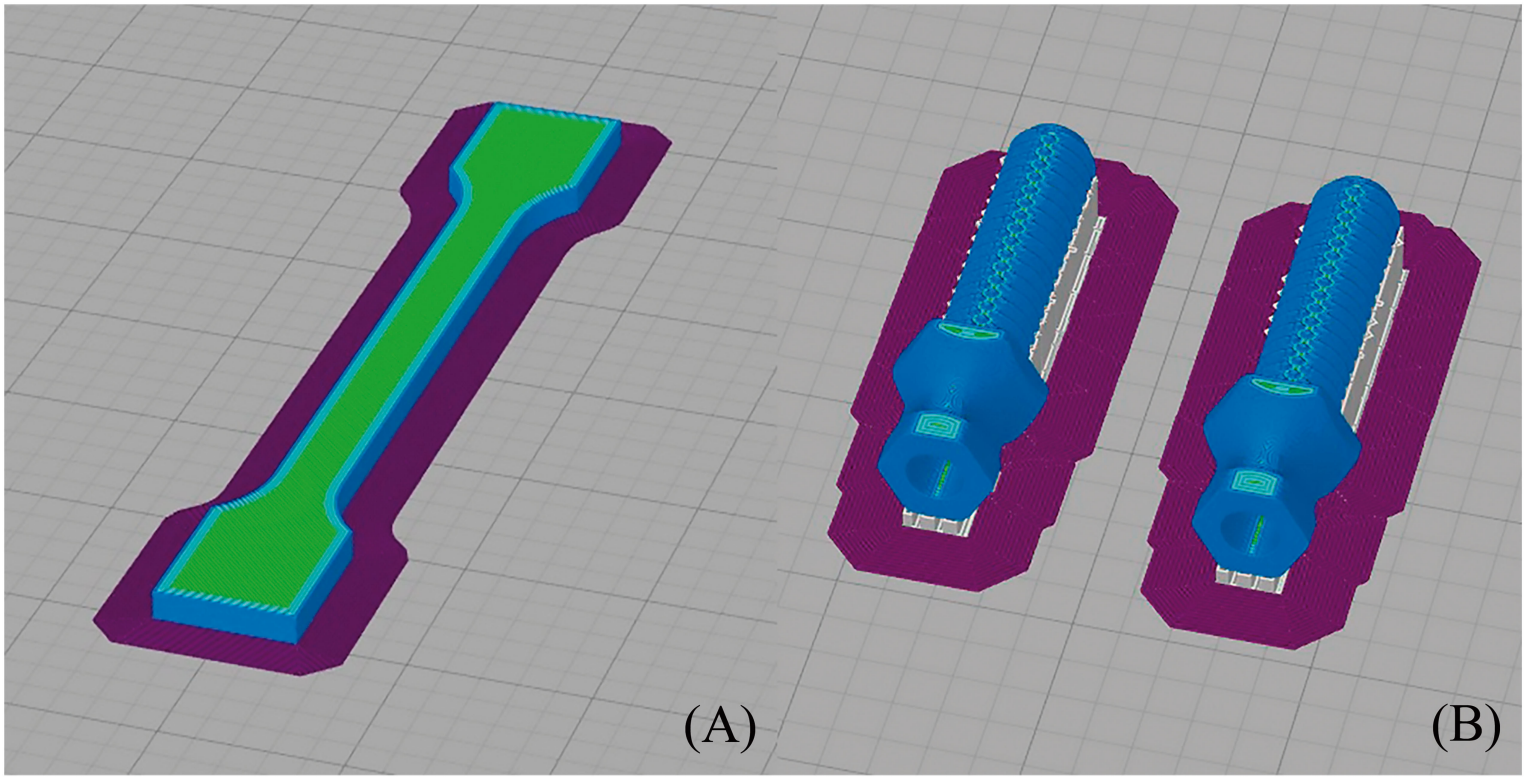
Printing simulation of **(A)** dog bone sample (its dimensions were 75 ± 0.5 mm length, 5 ± 0.2 mm width, and 3.5 ± 0.2 mm thickness, with a gauge length of 25 ± 0.5 mm) and **(B)** endoprosthesis (its dimensions were 16.54*50.21*14.72 mm^3^, which are width, length, and thickness of the printing bed, respectively) in Simplify 3D.

All 3D models were horizontally oriented with respect to the printing bed (36). The largest axes of the endoprostheses and tensile test samples with dog bone shapes were oriented parallel to the printing bed (Figure 2). Endoprosthesis dimensions were 16.54^*^50.21^*^14.72 mm^3^, which are x, y, and z of the printing bed, respectively. Also, the bending test samples were printed like rectangular bars, with the same printing orientation as the other 3D models. A horizontal orientation was carried out to take into account the main mechanical forces borne inside the medullary canal. The general printing parameters were constant for all 3D models, which are shown in Table 1. Also, the temperature parameters were the same for both 3D models: nozzle temperature of 410 °C, bed temperature of 130 °C, chamber temperature of 90 °C, and the cooling fan speed of 50%. All these parameters were set without variants between the 3D models because it has been proven that the mechanical properties of 3D-printed models varied due to printing conditions (21, 23, 37).

**Table 1:** Characteristics of FDM printings at Simplify 3D slicer. DB- and RB- samples, are tensile test and bending test samples.

		**Tibial endo-prosthesis**	**DB- and RB- samples**
N° Models each printing		2 (40 mm distance between them)	1
Extrusion multiplier		0.92	0.92
Layer height (mm)		0.05	0.05
Skirt	*Layer*	1	1
	*Skirt outlines*	15	15
Infill (%)		50	50
Speed	*Default (mm/s)*	30	30
	*Outline underspeed (%)*	50	50
	*Solid infill underspeed (%)*	80	80
	*Supports underspeed (%)*	80	80

Furthermore, immediately before each printing, a specific liquid fixative (Dimafix, DIMA 3D, Valladolid, Spain) for 3D printing was applied to the cold print bed for better adhesion of the material during printing. A preheat of the chamber and build bed temperatures were set and allowed to stabilize for at least for 30 min before starting any printing.

### Preparation of the Endoprosthesis-Tibia Construct

**Figure 3:**
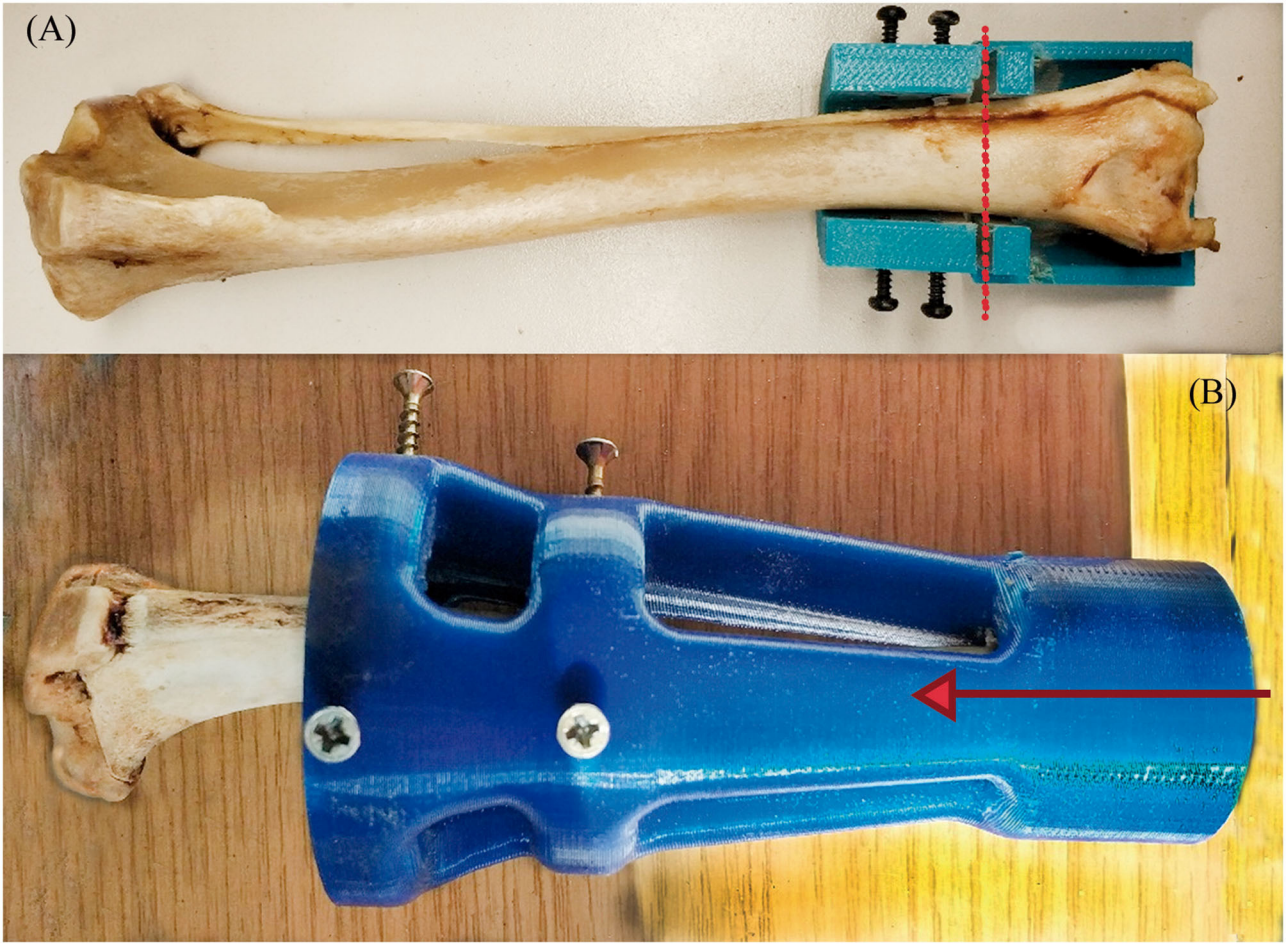
Transverse cut **(A)** and MC-concentric drill **(B)** guides. Both self-designed and printed at PLA. At **(A)**, the red dash line represents where the transversal cut was made on the distal tibia. At **(B)**, the red arrow represents the drilling direction of the MC to homogenize the MC diameter.

The endoprosthesis-tibia interface will be referred to as endoprosthesis-tibia construct in this article. The construct was assessed by mechanical tests. *Ex vivo* fresh tibiae were used to insert the endoprostheses. The 33 tibiae used for this study belonged to canine specimens (20.85 ± 1.25 kg BW) that were euthanized for reasons unrelated to this study. The longitudinal length of tibiae was 208.51 ± 40.27 mm. The fresh bones were immediately stored inside a vacuum bag in a freezer until the insertion of the endoprosthesis. Bones were perpendicularly cut to the longitudinal axes above the distal epiphysis. Immediately, the medullary canals of the tibiae were measured on the cut plane by a metric digital caliper. Tibiae with an average medullary canal diameter of 9.48 ± 0.29 mm were selected. This average medullary canal diameter was observed on the selected canine specimens. After measure, the medullary canal was drilled with a 9 mm bit to homogenize the canal. Two PLA 3D-printed self-designed surgical guides were used to make perpendicular cuts and to align drilling (Figure 3) (36). Insertion of the endoprosthesis was made by soft blows with a hammer. Later, an AISI 316 austenitic stainless steel threaded rod with a diameter of 6 mm was gently inserted using a locknut, with a length of 30 mm being left outside the PEEK sleeve. Cutting, drilling, and insertion were carried out by the same operator.

**Figure 4:**
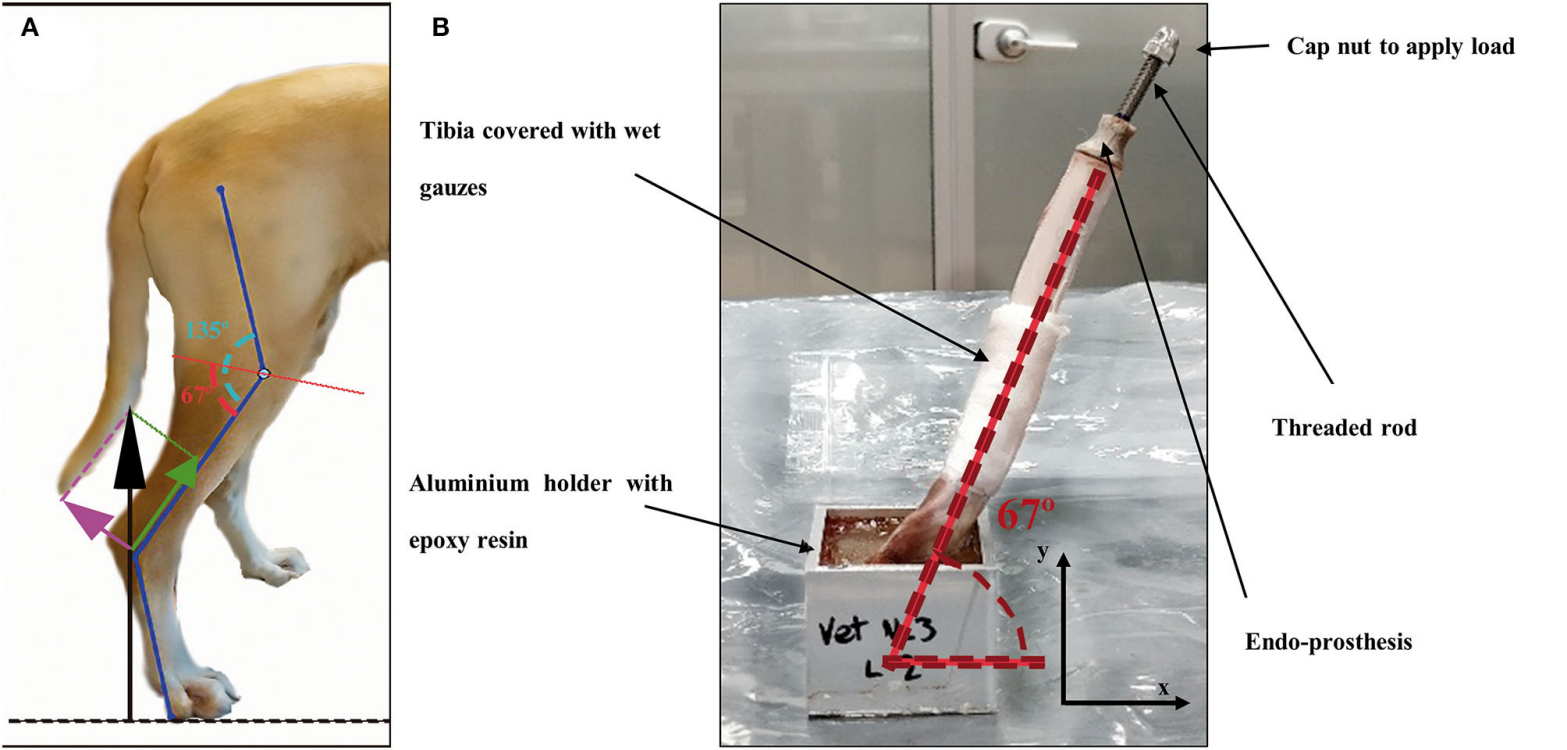
**(A)** Schematic figure of the decompounded ground reaction force (black arrow) on the canine tibia in compression (green arrow) and bending forces (pink arrow). Angle of the stifle joint (135°) was split in half by a red line as a reference for testing. **(B)** Endoprosthesis-tibia construct at 67° to the testing table for compression-bending and fatigue testing.

Once the endoprostheses were inserted, 25 tibiae were attached with a two-component epoxy resin (Resoltech 1050/1058S, Eguilles, France) in aluminum holders. Tibiae were cured in the holders at half the average angle of the stifle joint when the dog is standing (Figure 4A), as described by Holler et al. (38). The angle value of attachment was 67° of the tibial axis to the test table (Figure 4B) for compression-bending and fatigue tests. This angle is consistent with the average angle in maximum weight-bearing during trotting, acceleration, and galloping, as it has been described in the introduction section. The remaining eight tibiae were perpendicularly clamped to the test table for a pure compression test.

### Mechanical Testing

#### Mechanical Characterization of the 3D-Printed PEEK

The tensile properties of the 3D-printed PEEK were determined using tensile test samples with dog bone shapes (DB-samples) (39) (Figure 2A). The dimensions for each sample were 75 ± 0.5 mm in length, 5 ± 0.2 mm in width, and 3.5 ± 0.2 mm in thickness, with a gauge length of 25 ± 0.5 mm. All these measurements were according to the Spanish standard UNE 116005:2012 (39). Tests were performed according to ISO 527-1 (24) using the universal testing machine Elib 20W (SAE Ibertest Madrid, Spain) with a load cell of 2 kN, which operated at a crosshead speed of 1 mm/min.

A three-point bending load arrangement was used to determine the bending properties in accordance with ISO 178 (40). The tests were conducted using the universal testing machine IBTH/500 (Ibertest, Madrid, Spain) with a load cell of 500 N, which operated at a crosshead speed of 5 mm/min. For the bending tests, the samples were rectangular bars (RB-samples) with dimensions of 80.0 ± 0.1 mm length, 10.0 ± 0.1 mm width, and 4.0 ± 0.1 mm thickness [dimensions according to the Spanish standard UNE 116005:2012 (39)]. Eight samples were tested for each kind of test.

The results of the elastic properties were compared with those of canine tibiae, bulk PEEK, 316L stainless steel (316L-SS), and titanium (Ti) obtained from the literature (Table 2).

**Table 2:** Elastic properties of bulk PEEK, stainless steel, titanium, and canine tibia obtained from the literature.

**Material**	**Tensile strength (MPa)**	**Young's modulus (GPa)**	**Bending strength (MPa)**
Bulk PEEK (41)	97–117	3.76–3.95	105–116
316L-SS (41–43)	90–1,100	193–210	170–310
Ti (46,47)	240–1,100	110	130–1,280
Canine Tibia (44, 45)	107.7–199.12	8.85–15.59	142–193

#### Quasistatic Mechanical Testing of the Endoprosthesis

**Figure 5:**
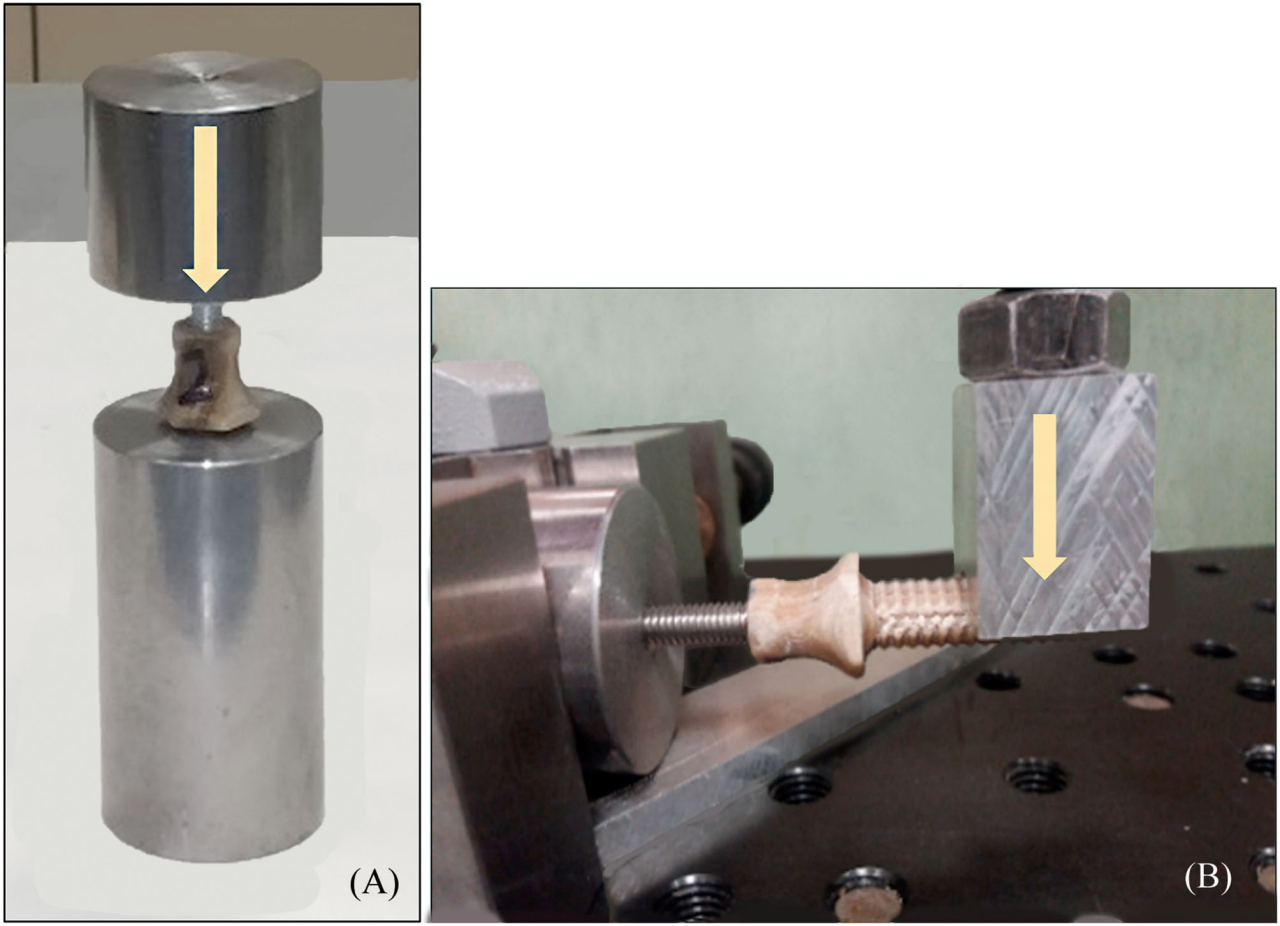
**(A)** Pure compression (endoprosthesis at vertical position) and **(B)** bending test (endoprosthesis at horizontal position) of the endoprosthesis. Yellow arrows represent the direction of the applied force.

Pure compression (Figure 5A) and bending (Figure 5B) tests were carried out onto endoprostheses themselves (E-samples) using the universal testing machine Elib 20W with a load cell of 2 kN, which operated at a crosshead speed of 500 mm/min. Five samples were tested for each kind of test. The aim of these tests was to find the weakest zone of the endoprosthesis and estimate if the mechanical strength of the endoprosthesis in the main load direction (vertical position) was enough to support the BW of the dog.

#### Pure Compression and Compression–Bending Testing of the Endoprosthesis-Tibia Construct

**Figure 6:**
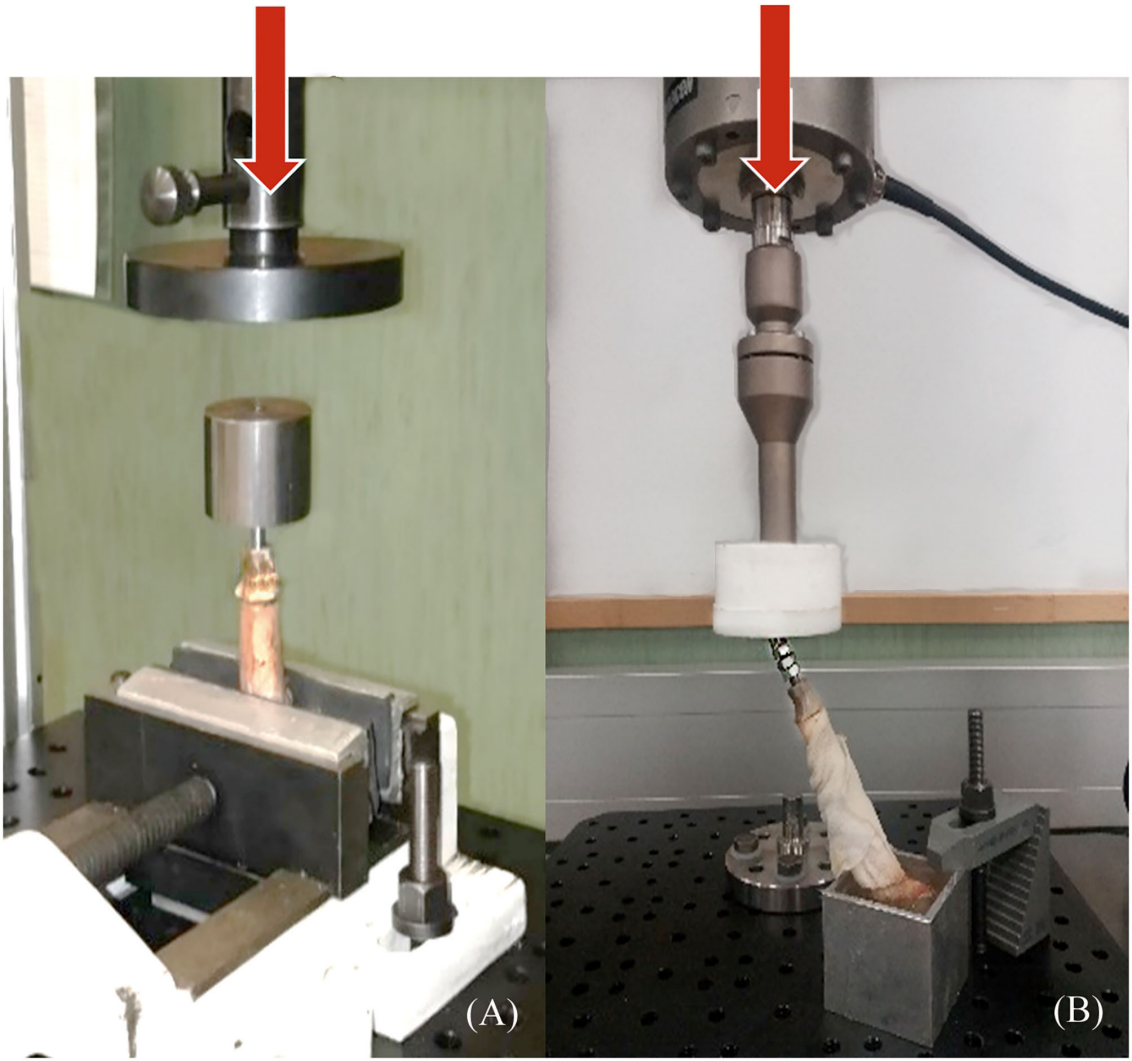
Pure compression **(A)** and compression-bending **(B)** tests of endoprosthesis-tibia construct. **(A)** samples were tested clamped directly to the bone, and **(B)** samples were potted at 67° to the ground before testing. Red arrows represent the direction of the applied force.

To obtain a more realistic result about the mechanical behavior of the endoprosthesis-tibia construct, samples (ET-samples) were prepared as described in section 2.4 above for pure compression (90°) and compression-bending test (67°). Each of the ET samples was assessed using the universal testing machine Elib 20 W (SAE Ibertest Madrid, Spain) with a load cell of 2 kN (Figure 6A). Tests were conducted under room conditions (22 ± 1 °C). Bones of ET-samples were covered with a gauze soaked in phosphate buffer solution (PBS) to maintain the correct pH and degree of humidity, simulating the *in vivo* conditions. Eight ET-samples were evaluated by pure compression testing (Figure 6A) and 15 ET-samples by compression-bending testing (Figure 6B) at a crosshead speed of 500 mm/min.

#### Fatigue Compression-Bending Testing of the Endoprosthesis-Tibia Construct

Fatigue tests are particularly important to study the long-term behavior of the endoprosthesis-tibia construct. The load to be supported by the tibia can be approximated by equation 1 (26, 30). For a theoretical 20 kg dog, if we consider the most unfavorable circumstances (dog at galloping), the maximum load to support would be approximately 300 N. This value was also considered as a working load to determine the FoS.


(1) $$\begin{array}{r} Maximum\;load\;\left(N\right)=1.5\;x\;DW\;\left(dog\;weight\;in\;kg\right)\;x\;9.8 \end{array}$$

Fatigue testing was conducted under room conditions (22 ± 1 °C) using an electrodynamic testing machine with a load cell of 3 kN (ElectroPlus E3000, Instron, Norwood, MA, USA). ET-samples were prepared as described in section 2.4 (Figure 6B) for compression-bending testing. These samples were assessed until failure or until 500,000 cycles, using a sinusoidal force cycle with a maximum load of 300 N and a load ratio of *R* = 0.1. A frequency of 2 Hz was used for the test, which is similar to the pacing frequency when the dog is trotting. Under these conditions, 10 ET-samples were evaluated.

### Statistical Analysis

Data from pure compression of E-samples and ET-samples tests were analyzed by analysis of variance (ANOVA) using the STATGRAPHICS program (XVII Centurion. Ver. 17.2.00, StatPoint, Inc., Herndon, VA, USA). Also, compression-bending data with or without fatigue tests were analyzed by analysis of variance (ANOVA). Significant differences were observed (*p* ≤ 0.05) by the *F*-test for both comparisons.

## Results

### Mechanical Testing of 3D-Printed PEEK Samples

Tensile strength, Young's modulus, and bending strength of the DB- and RB- samples, as described in section 2.5.1, were 36–52 MPa, 2.1–2.9 GPa, and 46–70 MPa, respectively. These results were compared with those of Ti, 316L-SS, bulk PEEK, and canine tibiae obtained from the literature. 3D-printed PEEK exhibited a reduction of tensile strength of 2- to 16-fold compared with the other materials. Comparing 3D-printed PEEK with bulk PEEK, the reduction was 2.5-fold. Young's modulus of 3D-printed PEEK was closer to that of bone than to that of metal. 3D-printed PEEK was 0.2-fold inferior to that of bone, while 316L-SS and Ti alloys exhibited Young's modulus 9- and 16-fold higher, respectively (Supplementary File 1).

3D-printed PEEK exhibited a reduction of bending strength of 1.9- to 12.2-fold compared with that of other materials. Additionally, its bending strength was closer to that of bone than to that of metal, especially compared to Ti.

### Mechanical Testing of the Endoprosthesis

#### Mechanical Testing of the Non-inserted Endoprosthesis

In all the bending tests, the endoprosthesis remained intact, while the steel rod showed plastic deformation. In the compression tests, the failure occurred between the umbrella and stem of the endoprosthesis, with a maximum load of 936 ± 199 N (Supplementary File 2).

#### Compression-Bending and Pure Compression Testing of the Endoprosthesis-Tibia Construct

**Figure 7:**
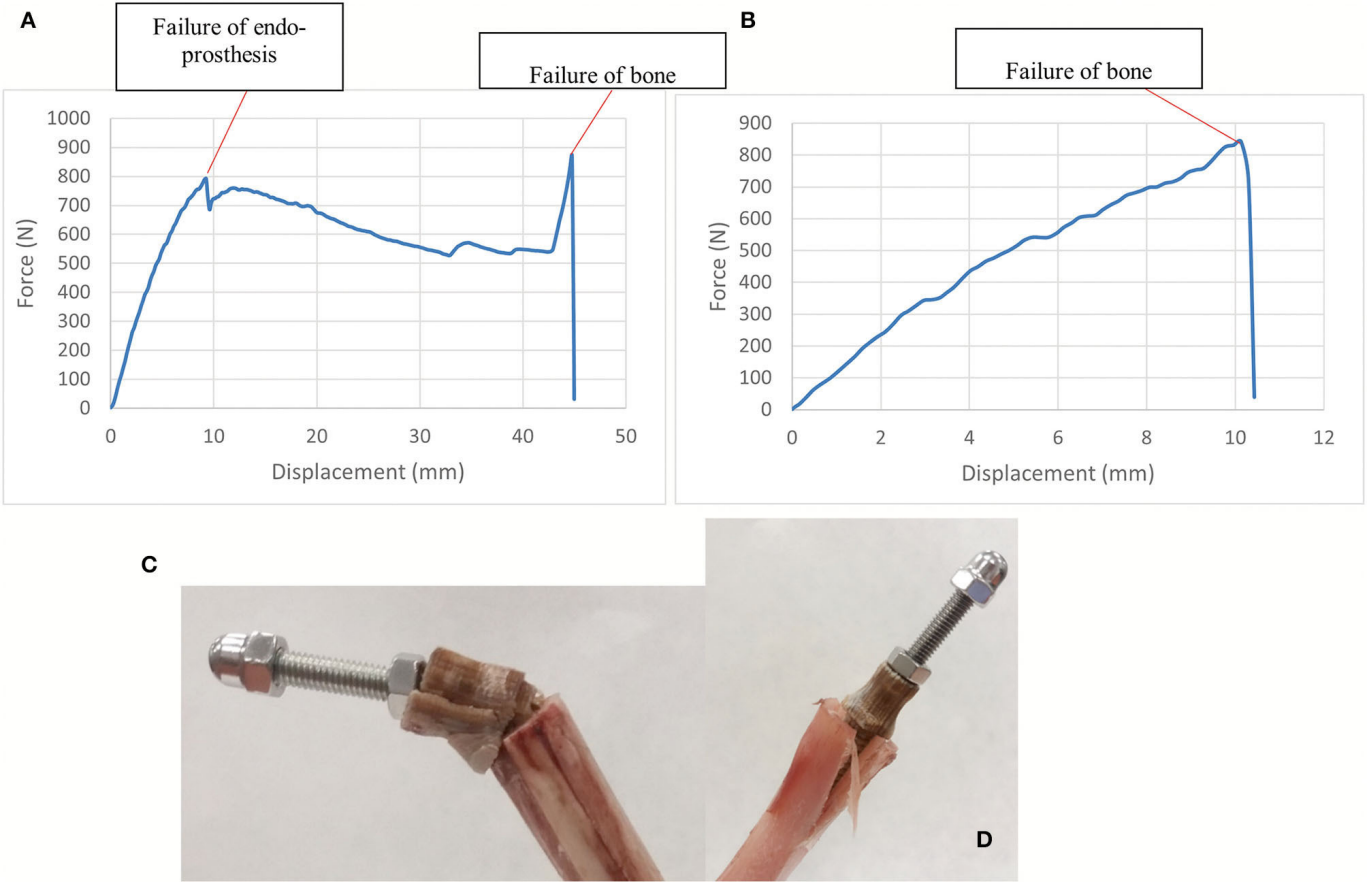
**(A,C)** show force-displacement curve and ET-samples after endoprosthesis failure in a long tibia. **(B,D)** show force-displacement curve and ET-samples after bone failure in a short tibia. Compression-bending test of the tibia-endoprosthesis constructs at 67° to the ground.

The compression-bending test (67°) was assessed for the 15 ET-samples (Supplementary File 3). The tested samples showed two different types of failure. In eight of them, the endoprosthesis broke, leading to a force-displacement graph with a first peak load due to endoprosthesis failure and a second peak that indicates the fracture of the tibia (Figures 7A,C). The bone failure occurred in the seven remaining samples, without any damage to the endoprosthesis after testing. In these cases, the force-displacement curves only showed the peak of the tibia fracture (Figures 7B,D). Considering only the breaking data of the eight endoprostheses, the average failure force is 785 ± 101 N. The calculated FoS was 2.6 ± 0.3 for ET-samples at the compression–bending test.

**Figure 8:**
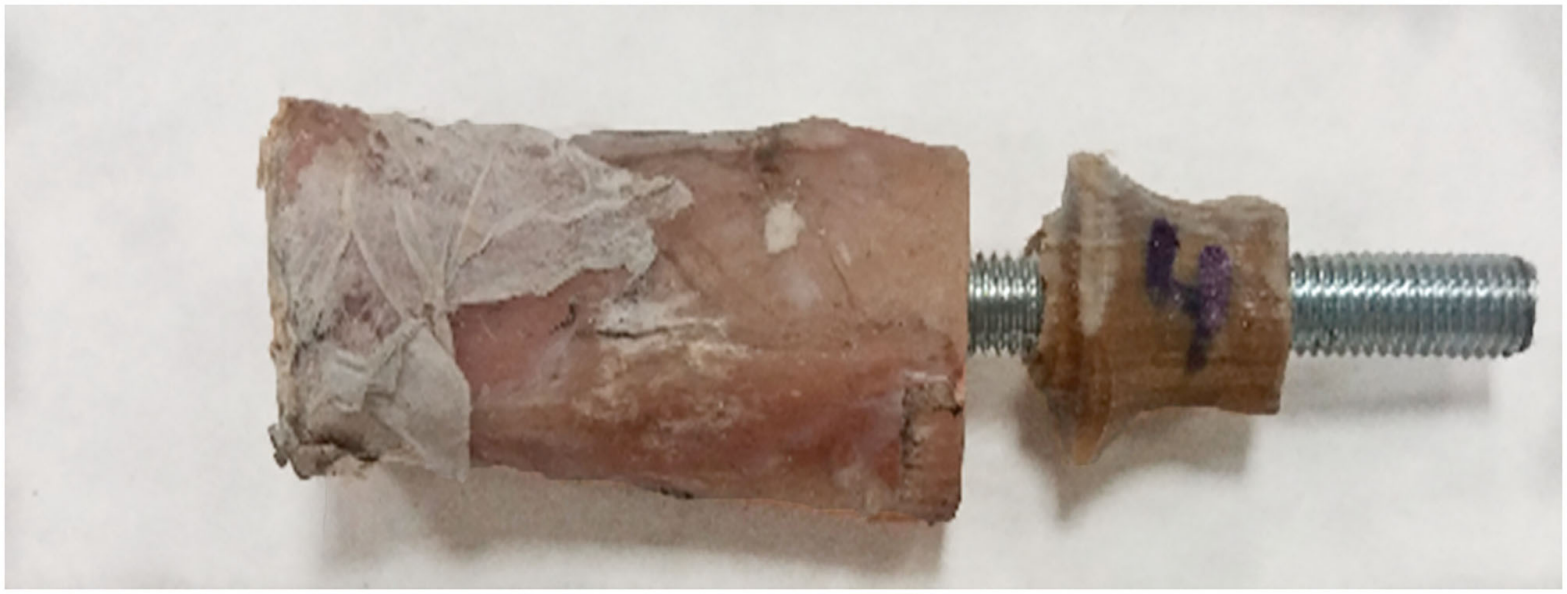
Failure example of the tibia-endoprosthesis constructs observed after pure compression test.

The results of the pure compression test reached a maximum force of 1,642 ± 447 N before failure (Supplementary File 2). For higher forces, the endoprostheses failed between the umbrella and distal tibia (Figure 8). The calculated FoS was 5.8 ± 1.4 for ET-samples at pure compression.

### Fatigue Testing of the Endoprosthesis-Tibia Construct at 67°

Upon fatigue test, the assessed ET-samples reached 500,000 cycles without failure. To analyze the effect of fatigue, the survivor samples were evaluated for failure by means of the quasistatic compression-bending test described in section 2.5.3. The samples could stand 764 ± 62 N upon static compression-bending test after fatigue before breaking (Supplementary File 3). No statically significant differences were observed between the maximum force at the compression–bending test with or without the previous fatigue test with a *p* ≤ 0.05. The calculated FoS was 2.5 ± 0.2 for ET-samples at the compression–bending test after fatigue.

## Discussion

The 3D-printed endoprosthetic element can withstand normal weight-bearing loads reported in dogs at walk and trot. Additionally, we demonstrate that the behavior of the construct with acute loading to failure is not significantly different if the endoprosthesis is pre-fatigued with 500,000 cycles of pre-loading. The PEEK part of the endoprosthesis bore higher flexion and compression forces than canine tibiae during galloping (26, 30). All obtained results during our tests were more than two times the vertical load that the endoprosthesis will have to support during the life of the canine. Thus, PEEK could be an interesting option for weight-bearing implants in quadrupedal animals because a single limb would support the entire weight of the animal.

The ET-samples showed a maximum load at failure of more than 2-fold the selected working load (300 N for a 20 kg dog) in the compression–bending test. These results are even more promising considering that we only used a wet canine tibia without the remaining skeletal system components of the pelvic limb. Besides, we are comparing compression-bending results with the force borne by the whole pelvic limb, which is another handicap for the studied endoprosthesis. In addition, it should be considered that the compression-bending tests were carried out in a standing-like position, imitating the canine stifle angle (38). It means that the endoprosthesis suffered bending and compression load, at the same time, during our compression-bending test. Taking into consideration that all tibiae had a medullary canal of 9 mm and the inserted endoprostheses had the same size, two types of failure have been described in the ET-samples during this test. In eight samples, failure occurred in both the prosthesis and bone, and in seven samples, the endoprosthesis remained intact while bone failure occurred. These failure modes could be explained due to the lengths of the assessed tibiae. Thus, if the ET-sample is considered as a cantilever, with more bone length, more bending moments would suffer the endo-prosthesis (9). Thus, in longer tibiae, both failure of bone and the PEEK part will be expected. We can confirm that these facts are disadvantages for the endoprosthesis because, during the bending test of the E-samples, the PEEK part remained intact while the steel rod showed plastic deformation.

The fatigue test shows the long-term behavior of a device. The results of fatigue and the following compression-bending test indicate that the endoprosthesis can withstand 500,000 cycles with a trotting-like frequency and the total BW during galloping without detriment of its strength. The fatigue parameters, cycles, and frequencies were set based on other papers where fatigue resistance was evaluated for different metal implants (32, 33) and assuming that trotting is the dog's pace with more step frequency. These test conditions were considered the most unfavorable scenario. No significant differences were observed between the maximum force at the compression–bending test with or without the previous fatigue test. Therefore, the FoS after fatigue indicated that the endoprosthesis designed for this work would not experience dangerous levels of load during normal activity.

Pure compression results confirm that force-bearing data from only pure compression of the E-samples or from pure compression of ET-samples are significantly different. The ET-samples could stand 1,642 ± 447 N on pure compression (5-fold increase in the dog's weight-bearing), in contrast to 936 ± 199 N in the E-samples. This means that the endoprosthesis has better results in an environment closer to a real one, such as using wet bones (46) as receptacles for the endoprosthesis. Bone is considered an anisotropic material that has different elastic behavior and strength according to the percentage of humidity, the forces applied, and the force speed application (46–48). Thus, achieving a test environment close to a real situation for medical application is essential for bone. Likewise, this increase in force-bearing results was because of the lateral compression exerted by the tibia during the endoprosthesis since part of the load applied to the endoprosthesis is transmitted to the tibia.

When a 3D printer is used to obtain solid pieces, the resultant material shows anisotropic behavior (49). Therefore, the 3D-printed PEEK properties will be different in each direction and will depend on the printing conditions. For this reason, mechanical characterization was carried out on 3D-printed PEEK samples (under the same printing conditions as the endoprostheses) to determine the mechanical properties of the PEEK material used for the endoprosthesis and to compare them with those of the tibiae. The differences observed in the mechanical properties between 3D-printed PEEK and bulk PEEK are due to the small spaces between the printed layers, which is typical in FDM technology, and to the infill parameter of 50% for the dog bone shape samples. This infill parameter was the same as that of the endoprostheses, which causes a drop in the mechanical properties (49) of 3D-printed PEEK. Nevertheless, its elasticity was still within canine cortical bone ranges and closer than those of metallic materials (21, 22). Young's modulus especially was in the same order of magnitude as that of bone. In addition, the tensile and bending strengths of 3D-printed PEEK are enough to guarantee that the endoprosthesis will deform similarly to the bone. Besides, the lower stiffness and strength of 3D-printed PEEK with respect to that of bone allows for a good transfer of the loads from the implant to the tibia, minimizing the probability of peri-implant bone loss due to stress-shielding (7, 10, 42). Also, using no annealed 3D-printed PEEK permits anisotropic material behavior (22, 23, 49). This makes the force transmission between PEEK and bone more interesting because putting two anisotropic materials in contact with a similar elastic modulus and transmitting the bearing forces through them could be a potential solution to the stress-shielding phenomenon. In addition, the interaction of PEEK and canine tibia can be evaluated by comprehending the mechanical characterization of 3D-printed PEEK, mechanical properties of the canine tibia, and force-bearing results of pure compression of ET-samples. The interaction of two materials with similar elastic properties, such as PEEK and canine tibia, made a similar deformation of both materials. Once the endoprosthesis is inserted on the tibia, it adapts its shape to the medullary canal. This deformation would allow the reduction or elimination of dead spaces between the internal part of the exo-endoprosthesis and the bone. This PEEK-bone interface seems to permit a better stress transmission, as pure compression data of ET-samples had been probed. This increment of stress transmission would reduce the possibilities of stress-shielding phenomenon in a real situation. Nonetheless, more studies about it should be done.

In conclusion, the evaluated endoprosthetic part of an exo-endoprosthesis can largely withstand a dog's weight during a galloping pace and at a higher frequency than this pace without detriment to its maximum weight-bearing. This makes the 3D-printed PEEK exo-endoprostheses a suitable mechanical choice for medical devices in veterinary medicine.

## Data Availability Statement

The original contributions presented in the study are included in the article/Supplementary Material, further inquiries can be directed to the corresponding author.

## Author Contributions

Conceptualization: RM-D, YB, JR-R, and JR-Q. Data curation, methodology, resources, and writing—original draft: RM-D, YB, and JR-R. Formal analysis and validation: RM-D and JR-R. Funding acquisition and project administration: JR-Q and SP-F. Investigation: RM-D, YB, JR-R, and EP. Software: RM-D, SP-F, EP, YB, and JR-R. Supervision: JR-Q, SP-F, and JR-R. Writing—review and editing: RM-D, YB, JR-R, and JR-Q. All authors contributed to the article and approved the submitted version.

## Funding

This work was supported by the regional government of Madrid, Spain [Grant IND2017/BMD-7726]. RM-D received a PhD fellowship from this grant. The regional government of Madrid was not involved in any decision about the development of this study.

## Conflict of Interest

RM-D and SP-F were employed by ABAX Innovation Technologies. The remaining authors declare that the research was conducted in the absence of any commercial or financial relationships that could be construed as a potential conflict of interest.

## Publisher's Note

All claims expressed in this article are solely those of the authors and do not necessarily represent those of their affiliated organizations, or those of the publisher, the editors and the reviewers. Any product that may be evaluated in this article, or claim that may be made by its manufacturer, is not guaranteed or endorsed by the publisher.

## Supplementary Material

Supplementary File 1 PEEK_tests.xlsx. Tested 3D-printed PEEK samples and their corresponding dimensions and strength results are shown. Characteristics of the tests, mean and standard deviation (SD) are also described.

Supplementary File 2 Compression_Tests.xlsx. Strength results of the endoprosthesis and bone-endoprosthesis assembly are described. Also, characteristics of the tests, mean, and standard deviation (SD) are also described.

Supplementary File 3 C-B_Tests.xlsx. Number of ET-samples and their corresponding results at compression-bending test before and after fatigue test are shown. Also, characteristics of the tests, mean, and standard deviation (SD) are also described.

## References

[1] Thesleff A Brånemark R Håkansson B Ortiz-Catalan M. Biomechanical characterisation of bone-anchored implant systems for amputation limb prostheses: a systematic review. Ann Biomed Eng. (2018) 46:377–91. 10.1007/s10439-017-1976-4 29327257 PMC5809556

[2] Fitzpatrick N Smith TJ Pendegrass CJ Yeadon R Ring M Goodship AE. Intraosseous Transcutaneous Amputation Prosthesis (ITAP) for limb salvage in 4 dogs. Vet Surg. (2011) 40:909–25. 10.1111/j.1532-950X.2011.00891.x 22092391

[3] Farrell BJ Prilutsky BI Kistenberg RS Dalton JF Pitkin M. An animal model to evaluate skin–implant–bone integration and gait with a prosthesis directly attached to the residual limb. Clin Biomech. (2014) 29:336–49. 10.1016/j.clinbiomech.2013.12.014 24405567 PMC3959271

[4] Drygas KA Taylor R Sidebotham CG Hugate RR Mcalexander H. Transcutaneous tibial implants: a surgical procedure for restoring ambulation after amputation of the distal aspect of the tibia in a dog. Vet Surg. (2008) 37:322–7. 10.1111/j.1532-950X.2008.00384.x 18564255

[5] DeVasConCellos P Balla VK Bose S Fugazzi R Dernell WS Bandyopadhyay A. Patient specific implants for amputation prostheses: design, manufacture and analysis. Vet Comp Orthop Traumatol. (2012) 25:286–96. 10.3415/VCOT-11-03-0043 22580779

[6] Golachowski A Al Ghabri MR Golachowska B Al Abri H Lubak M Sujeta M. Implantation of an intraosseous transcutaneous amputation prosthesis restoring ambulation after amputation of the distal aspect of the left tibia in an arabian Tahr (Arabitragus jayakari). Front Vet Sci. (2019) 6:182. 10.3389/fvets.2019.00182 31245397 PMC6579837

[7] Huiskes R Weinans H Rietbergen BV. The relationship between stress shielding and bone resorption around total hip stems and the effects of flexible materials. Clin Orthop Relat Res. (1992) 274:124–34. 10.1097/00003086-199201000-00014 1728998

[8] Wooley P Schwarz E. Aseptic loosening. Gene Ther. (2004) 11:402–7. 10.1038/sj.gt.3302202 14724679

[9] Sharir A Barak MM Shahar R. Whole bone mechanics and mechanical testing. Vet J. (2008) 177:8–17. 10.1016/j.tvjl.2007.09.012 17986396

[10] Sumner DR. Long-term implant fixation and stress-shielding in total hip replacement. J Biomech. (2015) 48:797–800. 10.1016/j.jbiomech.2014.12.021 25579990

[11] Kurtz SM. PEEK Biomaterials Handbook. 2nd ed. Amsterdam: Elsevier (2019). p. 465.

[12] Maté Sánchez de Val JE Gómez-Moreno G Pérez-Albacete Martínez C Ramírez-Fernández MP Granero-Marín JM Gehrke SA. Peri-implant tissue behavior around non-titanium material: experimental study in dogs. Ann Anat. (2016) 206:104–9. 10.1016/j.aanat.2016.03.005 27045596

[13] Wang N Xie H Xi C Zhang H Yan J. A study to compare the efficacy of polyether ether ketone rod device with titanium devices in posterior spinal fusion in a canine model. J Orthop Surg Res. (2017) 12:40. 10.1186/s13018-017-0543-x 28279204 PMC5345138

[14] Shimizu T Fujibayashi S Yamaguchi S Otsuki B Okuzu Y Matsushita T. *In vivo* experimental study of anterior cervical fusion using bioactive polyetheretherketone in a canine model. PLoS ONE. (2017) 12:e0184495. 10.1371/journal.pone.0184495 28886118 PMC5590956

[15] Atzeni E Salmi A. Economics of additive manufacturing for end-usable metal parts. Int J Adv Manuf Technol. (2012) 62:1147–55. 10.1007/s00170-011-3878-133268812

[16] Hopkinson N Dicknes P. Analysis of rapid manufacturing—using layer manufacturing processes for production. Proc Inst Mech Eng C J Mech Eng Sci. (2003) 217:31–9. 10.1243/09544060376255459616808067

[17] Popov VV Muller-Kamskii G Katz-Demyanetz A Kovalevsky A Usov S Trofimcow D. Additive manufacturing to veterinary practice: recovery of bony defects after the osteosarcoma resection in canines. Biomed Eng Lett. (2019) 9:97–108. 10.1007/s13534-018-00092-7 30956883 PMC6431339

[18] Bray JP Kersley A Downing W Crosse KR Worth AJ House AK. Clinical outcomes of patient-specific porous titanium endoprostheses in dogs with tumors of the mandible, radius, or tibia: 12 cases (2013–2016). J Am Vet Med Assoc. (2017) 251:566–79. 10.2460/javma.251.5.566 28828951

[19] Mendaza De Cal RM Peso Fernández S Rodríguez Quirós J. Endoprótesis a medida para huesos largos de animales (2020). Available online at: https://patentimages.storage.googleapis.com/0c/f4/54/d74e23ec379b45/ES2736410A1.pdf (accessed April 27, 2020).

[20] Steven Lathers Jeffrey La Belle. Osseointegratable prosthetic device and manufacturing method. US 2018/0049897 Al, 2018. p. 49.

[21] Arif MF Kumar S Varadarajan KM Cantwell WJ. Performance of biocompatible PEEK processed by fused deposition additive manufacturing. Mater Des. (2018) 146:249–59. 10.1016/j.matdes.2018.03.015

[22] Wu WZ Geng P Zhao J Zhang Y Rosen DW Zhang HB. Manufacture and thermal deformation analysis of semicrystalline polymer polyether ether ketone by 3D printing. Mat Res Innov. (2014) 18:S5-12–6. 10.1179/1432891714Z.000000000898

[23] Yang C Tian X Li D Cao Y Zhao F Shi C. Influence of thermal processing conditions in 3D printing on the crystallinity and mechanical properties of PEEK material. J Mat Process Technol. (2017) 248:1–7. 10.1016/j.jmatprotec.2017.04.027

[24] Plastics—Determination of Tensile Properties. Part 1: General Principles. Report No.: ISO 527-1:2019. Geneva: International Organization for Standardization (2019).

[25] Voss K Wiestner T Galeandro L Hässig MR Montavon PM. Effect of dog breed and body conformation on vertical ground reaction forces, impulses, and stance times. Vet Comp Orthop Traumatol. (2011) 24:106–12. 10.3415/VCOT-10-06-0098 21243175

[26] Walter RM Carrier DR. Ground forces applied by galloping dogs. J Exp Biol. (2007) 210:208–16. 10.1242/jeb.02645 17210958

[27] Colborne GR Walker AM Tattersall AJ Fuller CJ. Effect of trotting velocity on work patterns of the hind limbs of Greyhounds. Am J Vet Res. (2006) 67:1293–8. 10.2460/ajvr.67.8.1293 16881839

[28] Corbee RJ Maas H Doornenbal A Hazewinkel HAW. Forelimb and hindlimb ground reaction forces of walking cats: assessment and comparison with walking dogs. Vet J. (2014) 202:116–27. 10.1016/j.tvjl.2014.07.001 25155217

[29] Schwarz N Tichy A Peham C Bockstahler B. Vertical force distribution in the paws of sound labrador retrievers during walking. Vet J. (2017) 221:16–22. 10.1016/j.tvjl.2017.01.014 28283074

[30] Walter RM Carrier DR. Rapid acceleration in dogs: ground forces and body posture dynamics. J Exp Biol. (2009) 212:1930–9. 10.1242/jeb.023762 19483011

[31] Krotscheck U Todhunter RJ Nelson SA Sutter NB Mohammed HO. Precision and accuracy of ground reaction force normalization in a heterogeneous population of dogs: ground reaction force normalization. Vet Surg. (2014) 43:437–45. 10.1111/j.1532-950X.2014.12176.x 24702543

[32] Schmierer PA Smolders LA Zderic I Gueorguiev B Pozzi A Knell SC. Biomechanical properties of plate constructs for feline ilial fracture gap stabilization. Vet Surg. (2019) 48:88–95. 10.1111/vsu.13124 30422336

[33] Chao P Conrad BP Lewis DD Horodyski M. Pozzi A. Effect of plate working length on plate stiffness and cyclic fatigue life in a cadaveric femoral fracture gap model stabilized with a 12-hole 24 mm locking compression plate. BMC Vet Res. (2013) 9:125. 10.1186/1746-6148-9-125 23800317 PMC3704939

[34] Kim J Rietdyk S Breur GJ. Comparison of two-dimensional and three-dimensional systems for kinematic analysis of the sagittal motion of canine hind limbs during walking. Am J Vet Res. (2008) 69:1116–22. 10.2460/ajvr.69.9.1116 18764680

[35] Fischer MS Lilje KE. Dogs in motion. 2nd ed. Paderborn: VDH (2020).p. 207.

[36] Mendaza-DeCal R Peso-Fernandez S Rodriguez-Quiros J. Test of designing and manufacturing a polyether ether ketone endoprosthesis for canine extremities by 3D printing. Front Mech Eng. (2021) 7:693436. 10.3389/fmech.2021.693436

[37] Vaezi M Yang S. Extrusion-based additive manufacturing of PEEK for biomedical applications. Virtual Phys Prototyp. (2015) 10:123–35. 10.1080/17452759.2015.109705334077906

[38] Holler PJ Brazda V Dal-Bianco B Lewy E Mueller MC Peham C. Kinematic motion analysis of the joints of the forelimbs and hind limbs of dogs during walking exercise regimens. Am J Vet Res. (2010) 71:734–40. 10.2460/ajvr.71.7.734 20594074

[39] Manufacturing by additive of caps on plastics. Additive Manufacturing. Preparation of Test Pieces. Report No.: UNE 116005:2012. Madrid: Asociación Española de Normalización y Certificación (2012).

[40] Plastics—Determination of Flexural Properties. Report No.: ISO 178:2019. Geneva: International Organization for Standardization (2019).

[41] Swanson J. Ansys Granta EdoPack Software. Cambridge: Ansys Inc.

[42] Geetha M Singh AK Asokamani R Gogia AK. Ti based biomaterials, the ultimate choice for orthopaedic implants—a review. Prog Mater Sci. (2009) 54:397–425. 10.1016/j.pmatsci.2008.06.004

[43] Dewidar MM Yoon HC Lim JK. Mechanical properties of metals for biomedical applications using powder metallurgy process: a review. Met Mater Int. (2006) 12:193–206. 10.1007/BF03027531

[44] Saha S Martin DL Phillips A. Elastic and strength properties of canine long bones. Med Biol Eng Comput. (1977) 15:72–4. 10.1007/BF02441578 194127

[45] Kemp TJ Bachus KN Nairn JA Carrier DR. Functional trade-offs in the limb bones of dogs selected for running *versus* fighting. J Exp Biol. (2005) 208:3475–82. 10.1242/jeb.01814 16155220

[46] Lee KL Sobieraj M Baldassarri M Gupta N Pinisetty D Janal MN. The effects of loading conditions and specimen environment on the nanomechanical response of canine cortical bone. Mater Sci Eng C. (2013) 33:4582–6. 10.1016/j.msec.2013.07.018 24094163

[47] Sammarco GJ Burstein AH Davis WL Frankel VH. The biomechanics of torsional fractures: the effect of loading on ultimate properties. J Biomech. (1971) 4:113–7. 10.1016/0021-9290(71)90021-2 5119406

[48] Sielman E. Mechanical properties of long bones in dogs. Am J Vet Res. (1994) 55:3. 7978660

[49] Pu J McIlroy C Jones A Ashcroft I. Understanding mechanical properties in fused filament fabrication of polyether ether ketone. Addit Manuf . (2020) 37:101673. 10.1016/j.addma.2020.101673

